# A contribution to *Dongodytes* (s. str.) Deuve, 1993 (Coleoptera, Carabidae, Trechinae)

**DOI:** 10.3897/zookeys.772.25803

**Published:** 2018-07-06

**Authors:** Pingjing Yang, Sunbin Huang, Mingyi Tian

**Affiliations:** 1 Department of Entomology, College of Agriculture, South China Agricultural University, Wushan, Guangzhou, Guangdong, 510640, China

**Keywords:** aphaenopsian, cavernicolous, China, ground beetles

## Abstract

The hypogean genus *Dongodytes* Deuve, 1993, one of the most cave-adapted genera of ground beetles, is distributed in northern Guangxi, ranging from Mashan through Du’an, Bama, and Fengshan to Tian’e. Review of nominate subgenus Dongodytes Deuve, 1993, with new records for *D. fowleri* Deuve, 1993 and *D. grandis* Uéno, 1998 are provided. Meanwhile, *Dongodytes tonywhitteni* **sp. n.** is described from a limestone cave in Bama County. This interesting species is dedicated to the late Dr. Tony Whitten, a well-known cave biodiversity conservationist in Asia. A key to all species of *Dongodytes* (s. str.) is also provided.

## Introduction

One of the most important events of biospeleology in China was the discovery of the aphaenopsian beetle. During a survey of China-British Cave Exploration in 1988, a single and extremely modified beetle was discovered and collected by the English caver Simon Fowler in a limestone cave in Bama County, Northwest Guangxi Zhuang Autonomous Region. It was the first cavernicolous trechine species discovered in China and was treated as a new genus and species *Dongodytes fowleri* Deuve, 1993 (Deuve 1993). But *Sinaphaenops mirabilissimus* Uéno & Wang, 1991, another aphaenopsian beetle, was reported earlier though it was discovered three years later than the former (Uéno and Wang 1991).

Uéno (1998, 2005) described two *Dongodytes* species: *D. grandis* Uéno, 1998 from the cave Yuanyang Dong in Fengshan County and *D. giraffa* Uéno, 2005 from the cave Bahao Dong in Tian’e County. The former is very similar to the type species *Dongodytes fowleri*. However, *D. giraffa* is very peculiar by having narrow and extremely elongated pronotum which is nearly parallel-sided throughout and bearing discal setae, evident serrated along elytral lateral margins on shoulder areas and unmodified protarsomeres in male, that it should be a member of another lineage instead of *Dongodytes* (s. str.).

Several years later, Tian (2011) and Tian et al. (2014) added nine species of *Dongodytes* from caves in Du’an-Dahua Karst. Five of them (*D. deharvengi* Tian, 2011, *D. jinzhuensis* Tian, Yin & Huang, 2014, *D. brevipenis* Tian, Yin & Huang, 2014, *D. inexpectatus* Tian, Yin & Huang, 2014, and *D. yaophilus* Tian, Yin & Huang, 2014) are members of subgenus Dongodytodes Tian, 2011. The other four (*D. baxian* Tian, 2011, *D. elongatus* Tian, Yin & Huang, 2014, *D. troglodytes* Tian, Yin & Huang, 2014, and *D. lani* Tian, Yin & Huang, 2014) were treated as members of the nominate subgenus. Indeed, the above four species are very different from *Dongodytes fowleri* and relative species by having a particular type of male genitalia, which is very short and stout in the median lobe of aedeagus and very large in the basal orifice (Tian et al. 2014). According our un-published results of molecular analysis, we presume that they belong to another lineage other than *Dongodytes* (s. str.) and will be treated in our next paper.

The aim of this short paper is to provide new records for both the known *Dongodytes* (s. str.) species, and to describe a new species from Bama County.

## Material and methods

The beetle material for this study was collected with the naked eye using an aspirator in caves and kept in 50% ethanol. One individual of each species was preserved in 95% ethanol for molecular analysis. Other cave beetles used for comparing were dried and mounted specimens. All studied specimens are deposited in the insect collection of South China Agricultural University, Guangzhou, China (SCAU).

Dissections and observations were made under a Leica S8AP0 microscope. Dissected genital pieces, including the median lobe and parameres of the aedeagus, were glued onto small transparent plastic plates and pinned under the specimen. Habitus pictures were taken by means of the Keyence VHX-5000 digital microscope. Genitalia pictures were taken using the Canon EOS 40D camera connected to the Zeiss AX10 microscope, and then stacked and processed in the Adobe Photoshop CC software. Distribution maps were created by using Mapinfo software.

The length of the body was measured from the apex of the right mandible (in open position); the width of the body was taken as the maximum width of the elytra.

Abbreviations of other measurements used in the text are as follows:

**HLm** length of head including mandibles, from apex of right mandible to occipital suture

**HLl** length of head excluding mandibles, from front of labrum to occipital suture

**HW** maximum width of head

**PrL** length of prothorax, along the median line

**PnL** length of pronotum, as above

**PrW** maximum width of prothorax

**PnW** maximum width of pronotum

**PfW** width of pronotum at front

**PbW** width of pronotum at base

**EL** length of elytra, from base of scutellum to elytral apex

**EW** maximum width of combined elytra

## Taxonomy

### Genus *Dongodytes* Deuve, 1993

#### Subgenus Dongodytes

Taxon classificationAnimaliaColeopteraCarabidae

(s. str.) Deuve, 1993

Dongodytes Deuve, 1993: 292; Uéno 1998: 4; Tian et al. 2014: 73

##### Type species.

*Dongodytes fowleri* Deuve, 1993

##### Range.

**Figure 1.**
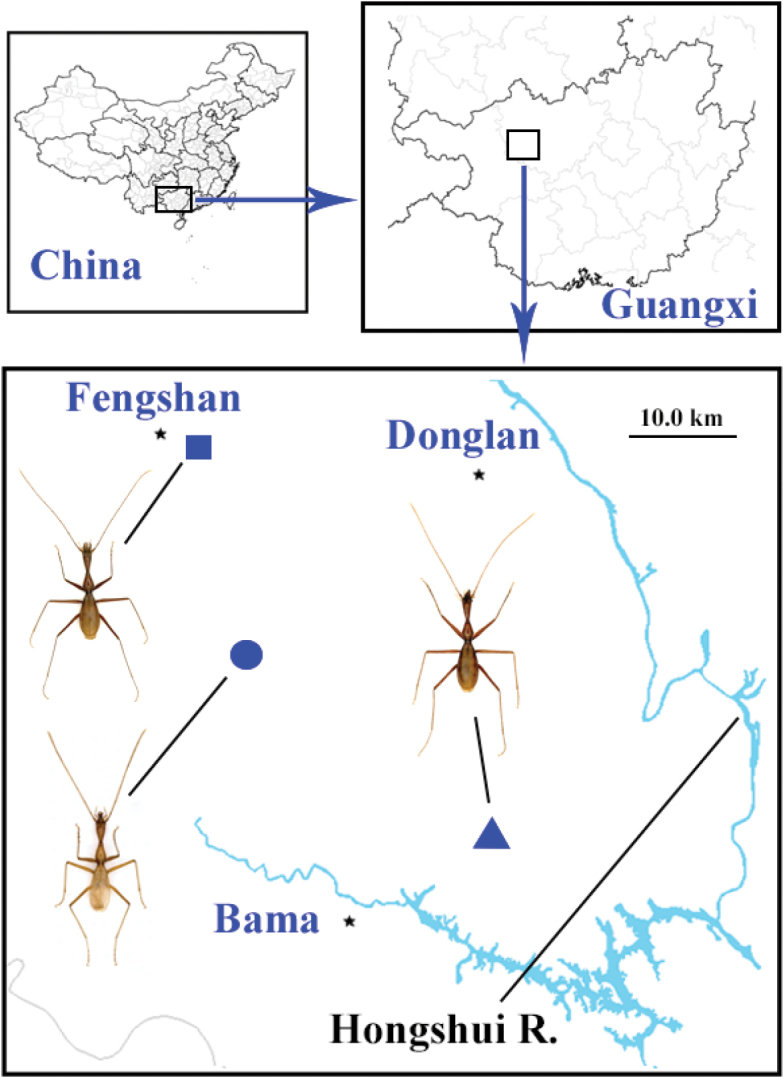
Range of the subgenus Dongodytes (s. str.) Deuve, 1993 (square = Dongodytes (s. str.) grandis, circle = Dongodytes (s. str.) fowleri, and triangle = Dongodytes (s. str.) tonywhitteni sp. n.)

Northwest Guangxi (Fengshan and Bama Counties) (Fig. 1).

#### Dongodytes (s. str.) fowleri

Taxon classificationAnimaliaColeopteraCarabidae

Deuve, 1993

**Figure 2.**
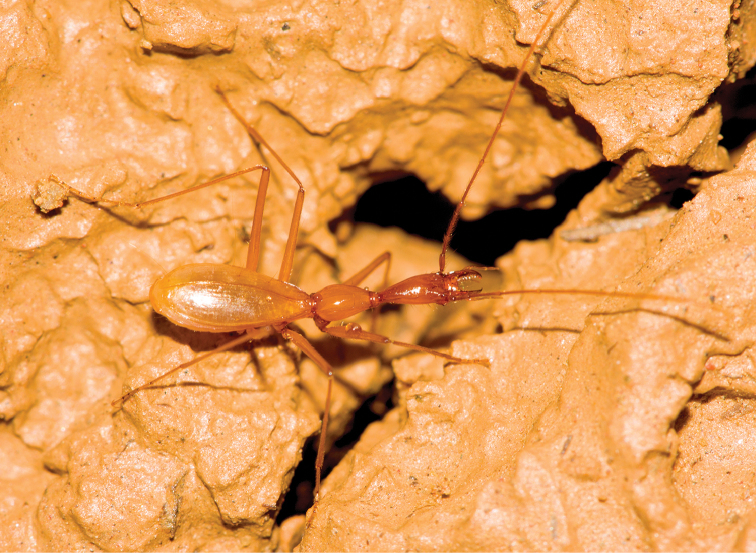
A living exemplar of Dongodytes (s. str.) fowleri Deuve, 1993.

Figs 1 , 2 , 5b , 6b

Dongodytes (s. str.) fowleri Deuve, 1993: 292; Uéno 1998: 8.

##### Type locality.

Jiabao Dong cave, Bama County.

##### Material.

1 female, Bama: Jiazhuan: Xingren: Jiabao: cave Xiaoshui Dong, 24°19'39.05"N/107°05'16.96"E, 347 m in altitude, 2017-III-11, leg. Sunbin Huang, Pingjing Yang & Dianmei Wang.

##### Distribution.

Guangxi (Bama) (Fig. 1). Known from two caves, Jiabao Dong (Uéno, 1998) and Xiaoshui Dong. The latter is a new locality for D. (s. str.) fowleri. It is ca. one kilometre from Jiabao Dong, and it has a large and dry chamber near the entrance which is ca. 10 m high and 8 m wide. The single specimen was discovered in a wet place on the right side of the second chamber which is ca. 100 m distant from the first one.

#### Dongodytes (s. str.) grandis

Taxon classificationAnimaliaColeopteraCarabidae

Uéno, 1998

**Figure 3.**
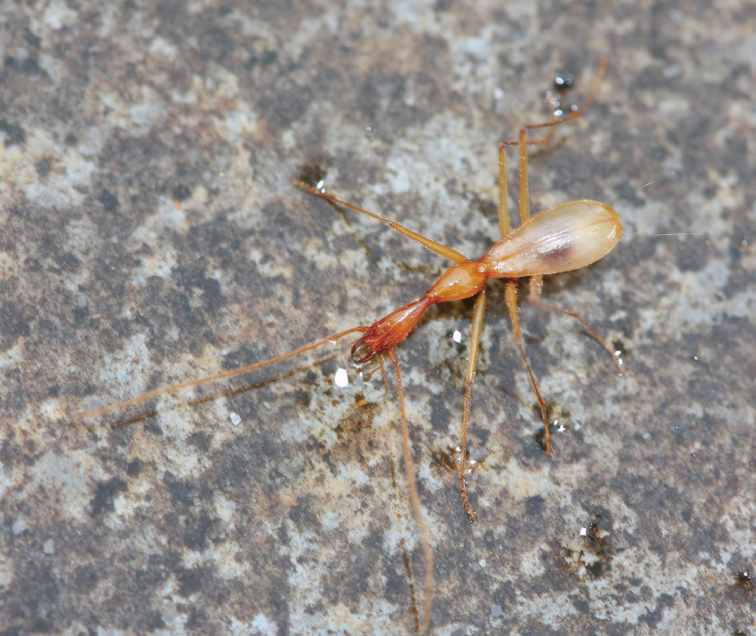
A living exemplar of Dongodytes (s. str.) grandis Uéno, 1998.

Figs 1 , 3 , 5c , 6c , 7c, d

Dongodytes (s. str.) grandis Uéno, 1998: 12.

##### Type locality.

Yuanyang Dong cave, Fengshan County.

##### Material.

1 male, Fengshan: Fengcheng: Fenghuang: cave Yuanyang Dong, 24°32'20.76"N/107°04'04.01"E, 691 m, 2015-VIII-3, leg. Xinhui Wang, Jujian Chen & Mingruo Tang; 1 male, 1 female, same cave, 2017-I-20, leg. Mingyi Tian & Jingli Cheng.

##### Distribution.

Guangxi (Fengshan) (Fig. 1). Known only from the cave Yuanyang Dong.

#### Dongodytes (s. str.) tonywhitteni sp. n.

Taxon classificationAnimaliaColeopteraCarabidae

http://zoobank.org/5E6380C9-E942-4360-AD80-DD09CC966956

**Figure 4.**
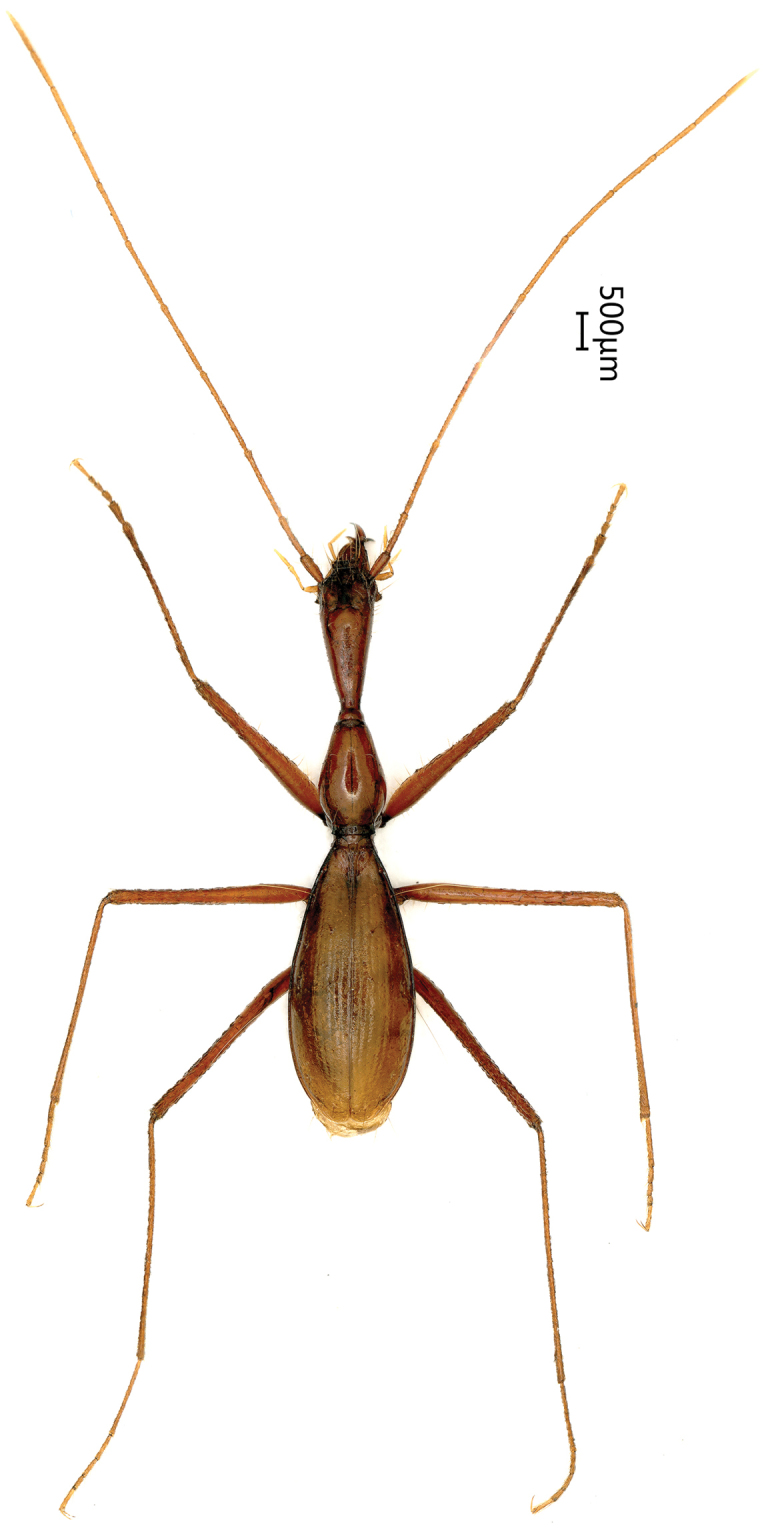
Habitus of *Dongodytes (s. str.) tonywhitteni* sp. n.

**Figure 5.**
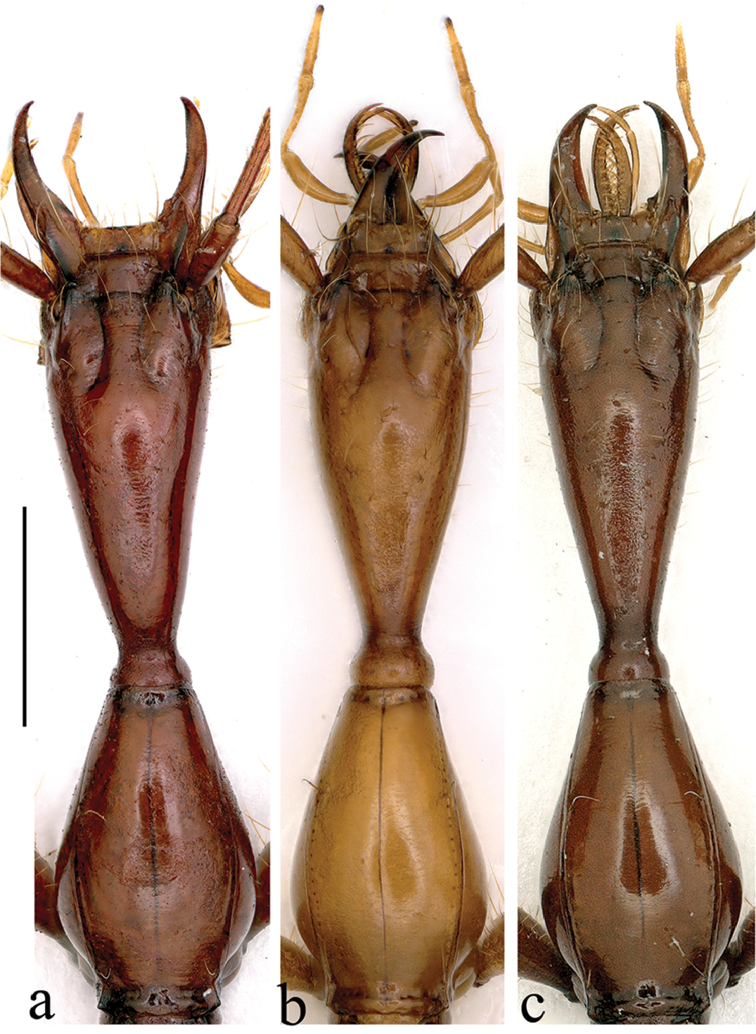
Head and pronotum of *Dongodytes* (s. str.) species **a** *D. tonywhitteni* sp. n. **b** *D. fowleri* **c** *D. grandis*. Scale bar: 1 mm.

**Figure 6.**
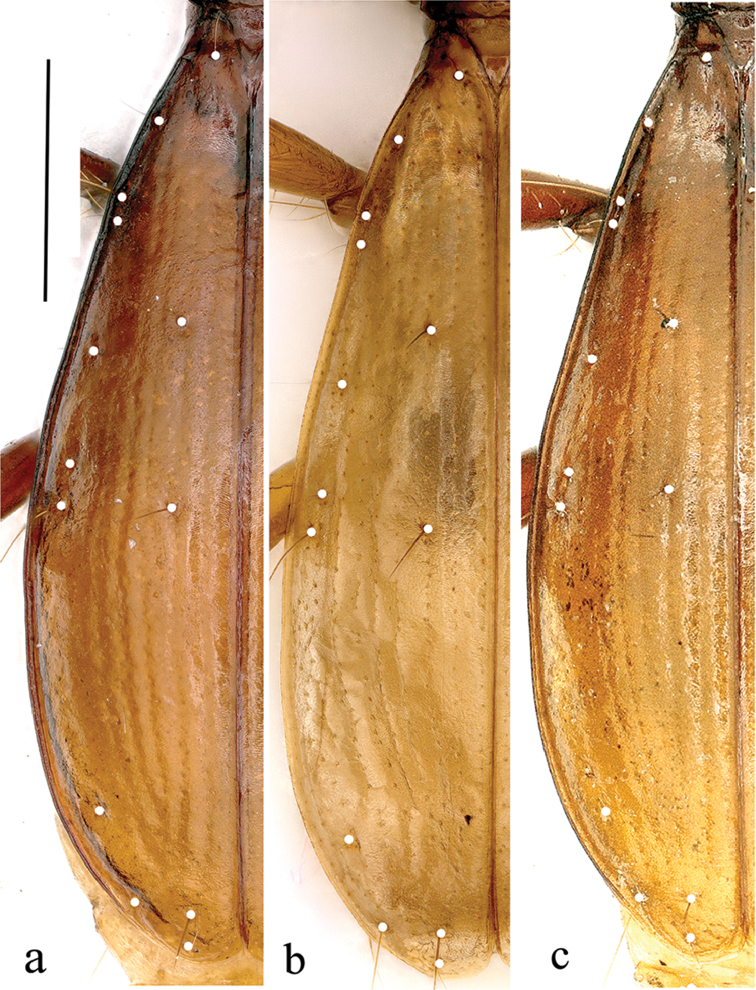
Left elytron of *Dongodytes* (s. str.) species, chaetotaxy shown by white points **a** *D. tonywhitteni* sp. n. **b** *D. fowleri* **c** *D. grandis*. Scale bar: 1 mm.

**Figure 7.**
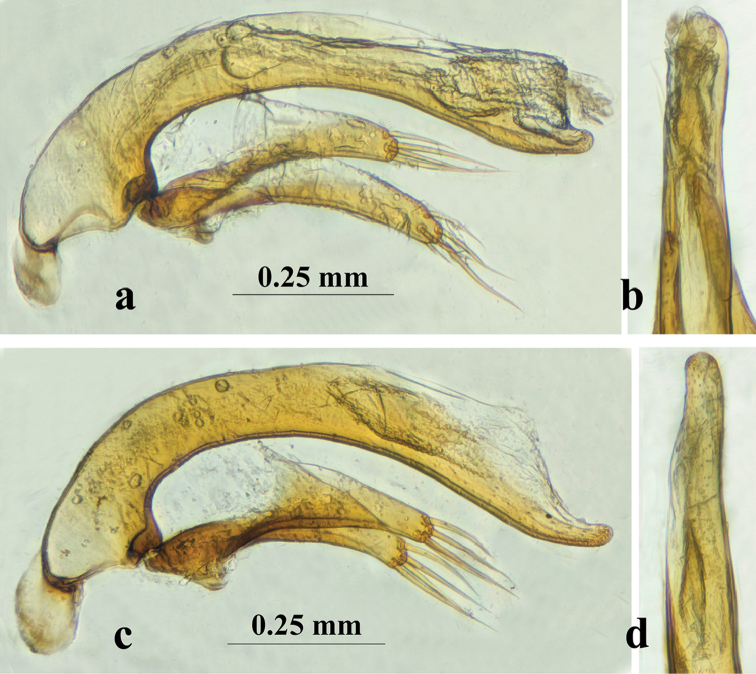
Male genitalia of *Dongodytes* (s. str.), median lobe and parameres (lateral view) and apical lobe (dorsal view) **a, b** *D. tonywhitteni* sp. n. **c, d** *D. grandis*.

Figs 1 , 4 , 5a , 6a , 7a, b

##### Material.

Holotype: male, Guangxi: Hechi: Bama: Fenghuang: Dena: Cave Nonggong Dong, 24°11'26.28"N / 107°23'41.49"E, 439 m in altitude, 2015-VII-31, leg. Xinhui Wang, Mingruo Tang & Jujian Chen leg., in SCAU; paratypes: 1 male and 2 females, ibid, in SCAU.

##### Diagnosis.

Large cave beetles, eyeless and depigmented, with very elongated body and appendages, antennae extending beyond apices of elytra, fore part of the body longer than elytra, protarsomere I slightly denticulate inwards at apices in male.

##### Description.

Length: 7.9–8.1 mm; width: 1.5–1.6 mm. Habitus as in Fig. 4.

Wholly reddish brown, with pale mouthparts palps and tarsi; slender and elongated body with very thin and long appendages, of which antennae evidently extending over apex of elytra. Smooth and glabrous on upper body surface but sparsely setose on head. Fore body longer (with mandibles) ((HLm+PrL)/EL = 1.12) or slightly shorter (excluding mandibles) ((HLl+PrL)/EL = 0.98) than elytra.

*Head* thin and very elongated, subconical but wider than that of both *D. fowleri* and *D. grandis*; much longer than wide, HLm/HW = 3.7, HLl/HW = 2.9; much longer than prothorax, HLm/PrL = 1.71, HLl/PrL = 1.35; narrower than prothorax and pronotum, HW/PrW = 0.78, HW/PnW = 0.94; presence of two pairs of supraorbital pores, genae more widened than in both *D. fowleri* and *D. grandis*; labial suture traceable; mentum bisetose, concave basally; mentum tooth small and short, simple at tip; submentum 6-setose; palps very thin and slender; maxilla palpomeres III and IV glabrous; labial palp II bisetose on inner margin, 1.5 times longer than palp III which is glabrous; suborbital pores nearer neck than submentum. Antennae thin and very long, much longer than whole body including mandibles, extending over elytral apices from apical part of antennomere IX.

*Prothorax* elongated, shorter than head, 1.67 times longer than wide, evidently tumid on propleura, widest at approximately 1/3 from base. Pronotum narrow and elongated, evidently wider than in both *D. fowleri* and *D. grandis*, twice as long as wide, PnL/PnW = 2.03; slightly wider than head, PnW/HW = 1.07; base wider than front (PbW/PfW = 1.6); widest at a little behind middle; lateral margins strongly sinuate before hind angles which are acute and sharp; anterior latero-marginal setae at approximately the apical 1/3, basal ones before hind angles, at exactly the sinuated points. Scutellum small.

*Elytra* very elongated ovate, similar in both *D. fowleri* and *D. grandis* in shape and chaetotaxy, with well-marked lateral borders; EL/EW = 2.22, EL/PnL = 2.42, EW/PnW= 1.82.

*Legs* thin and very long, protibiae smooth, without longitudinal sulci, protarsomere I evidently longer than wide.

*Ventrite IV-VI* each with three pairs of paramedian setae; in female, IV and V each with a pair of setae, VI with two pairs in male; VII quadrisetose in female, bisetose in male.

*Male genitalia* (Fig. 7a, b): Aedeagus moderately sclerotized, median lobe similar to *D. grandis* Uéno, 1998 (Fig. 7c, d), but less curved ventrally, base a little stouter, with a smaller and narrower sagittal aileron, apex less reflexed, apical lobe wider, left paramere bearing four or five long setae at apex.

##### Remarks.

*Dongodytes tonywhitteni* sp. n. is closer to *D. grandis* than to *D. fowleri* Deuve, 1993 because both former two species have slenderer and longer aedeagus (Fig. 7), although Nonggong Dong cave is closer to Jiabao Dong or Xiaoshui Dong (the localities of *D. fowleri* Deuve, 1993) than to Yuanyang Dong (the locality of *D. grandis* Uéno, 1998) (Fig. 1). However, there are several differences in genital structures (mentioned above) between them. The striking morphological character of *D. tonywhitteni* sp. n. is that lateral sides of pronotum is abruptly and strongly sinuate before the acute hind angles (Fig. 5a), versus gently sinuate before hind angles which are obtuse or more or less rectangular in both known species (Fig. 5b, c).

##### Etymology.

The name of this new species is dedicated to the late Dr. Tony Whitten (Fauna & Flora International, Cambridge, UK.), a famous biological conservationist in China and Southeast Asia who provided crucial support for cave biodiversity study in China.

##### Distribution.

China (Guangxi: Bama County). Known only from the type locality, cave Nonggong Dong (Fig. 1).

The entrance of cave Nonggong Dong is largely opened and surrounded by bushes. Its length remains unknown. There is a large room near entrance, and it is very humid and muddy. An underground stream goes along the main passage which is very deep, and some parts of the cave are interrupted by vertical shafts. The trechine beetles were collected in dark areas ca. 10–30 metres away from the entrance. Other cave animals found also in this cave were millipedes, crickets, woodlice, and bats.

### Key to species of the subgenus Dongodytes (s. str.)

**Table:** 

1	Pronotum widened, lateral margins suddenly and deeply sinuate just before hind angles which are well-marked and acute (Fig. 5a)	**D. (s. str.) tonywhitteni sp. n.**
–	Pronotum narrowed, lateral margins slightly sinuate before hind angles which are not well-marked and blunt (Fig. 5b, c)	**2**
2	Protarsomere 4 stout, slightly longer than wide, lateral sides of pronotum invisible from above in apical fifth, hind angles of pronotum sharp	**D. (s. str.) fowleri Deuve**
–	Protarsomere 4 slender, evidently longer than wide, lateral sides of pronotum invisible from above only near upper margin, hind angles of pronotum obtuse	**D. (s. str.) grandis Uéno**

## Supplementary Material

XML Treatment for Dongodytes

XML Treatment for Dongodytes (s. str.) fowleri

XML Treatment for Dongodytes (s. str.) grandis

XML Treatment for Dongodytes (s. str.) tonywhitteni
